# Effect of Psychological and Medication Therapies for Insomnia on Daytime Functions: A Randomized Clinical Trial

**DOI:** 10.1001/jamanetworkopen.2023.49638

**Published:** 2023-12-28

**Authors:** Charles M. Morin, Si-Jing Chen, Hans Ivers, Simon Beaulieu-Bonneau, Andrew D. Krystal, Bernard Guay, Lynda Bélanger, Ann Cartwright, Bryan Simmons, Manon Lamy, Mindy Busby, Jack D. Edinger

**Affiliations:** 1 Centre de Recherche CERVO/Brain Research Center, École de Psychologie, Université Laval, Quebec City, Quebec, Canada; 2 Li Chiu Kong Family Sleep Assessment Unit, Department of Psychiatry, Faculty of Medicine, The Chinese University of Hong Kong, Hong Kong SAR, China; 3 Department of Psychiatry and Behavioral Services, University of California, San Francisco; 4 University of Colorado, Denver; 5 National Jewish Health, Denver, Colorado; 6 Duke University Medical Center, Durham, North Carolina

## Abstract

**Importance:**

Daytime functional impairments are the primary reasons for patients with insomnia to seek treatment, yet little is known about what the optimal treatment is for improving daytime functions and how best to proceed with treatment for patients whose insomnia has not remitted.

**Objectives:**

To compare the efficacy of behavioral therapy (BT) and zolpidem as initial therapies for improving daytime functions among patients with insomnia and evaluate the added value of a second treatment for patients whose insomnia has not remitted.

**Design, Setting, and Participants:**

In this sequential multiple-assignment randomized clinical trial conducted at institutions in Canada and the US, 211 adults with chronic insomnia disorder were enrolled between May 1, 2012, and December 31, 2015, and followed up for 12 months. Statistical analyses were performed on an intention-to-treat basis in April and October 2023.

**Interventions:**

Participants were randomly assigned to either BT or zolpidem as first-stage therapy, and those whose insomnia had not remitted received a second-stage psychological therapy (BT or cognitive therapy) or medication therapy (zolpidem or trazodone).

**Main Outcomes and Measures:**

Study outcomes were daytime symptoms of insomnia, including mood disturbances, fatigue, functional impairments of insomnia, and scores on the 36-item Short-Form Health Survey (SF-36) physical and mental health components.

**Results:**

Among 211 adults with insomnia (132 women [63%]; mean [SD] age, 45.6 [14.9] years), 104 were allocated to BT and 107 to zolpidem at the first stage. First-stage treatment with BT or zolpidem yielded significant and equivalent benefits for most of the daytime outcomes, including depressive symptoms (Beck Depression Inventory-II mean score change, −3.5 [95% CI, −4.7 to −2.3] vs −4.3 [95% CI, −5.7 to −2.9]), fatigue (Multidimensional Fatigue Inventory mean score change, −4.7 [95% CI, −7.3 to −2.2] vs −5.2 [95% CI, −7.9 to −2.5]), functional impairments (Work and Social Adjustment Scale mean score change, −5.0 [95% CI, −6.7 to −3.3] vs −5.1 [95% CI, −7.2 to −2.9]), and mental health (SF-36 mental health subscale mean score change, 3.5 [95% CI, 1.9-5.1] vs 2.5 [95% CI, 0.4-4.5]), while BT produced larger improvements for anxiety symptoms relative to zolpidem (State-Trait Anxiety Inventory mean score change, −4.1 [95% CI, –5.8 to –2.4] vs −1.2 [95% CI, −3.0 to 0.5]; *P* = .02; Cohen *d* = 0.55). Second-stage therapy produced additional improvements for the 2 conditions starting with zolpidem at posttreatment in fatigue (Multidimensional Fatigue Inventory mean score change: zolpidem plus BT, −3.8 [95% CI, −7.1 to −0.4]; zolpidem plus trazodone, −3.7 [95% CI, −6.3 to −1.1]), functional impairments (Work and Social Adjustment Scale mean score change: zolpidem plus BT, −3.7 [95% CI, −6.4 to −1.0]; zolpidem plus trazodone, −3.3 [95% CI, −5.9 to −0.7]) and mental health (SF-36 mental health subscale mean score change: zolpidem plus BT, 5.3 [95% CI, 2.7-7.9]; zolpidem plus trazodone, 2.0 [95% CI, 0.1-4.0]). Treatment benefits achieved at posttreatment were well maintained throughout the 12-month follow-up, and additional improvements were noted for patients receiving the BT treatment sequences.

**Conclusions and Relevance:**

In this randomized clinical trial of adults with insomnia disorder, BT and zolpidem produced improvements for various daytime symptoms of insomnia that were no different between treatments. Adding a second treatment offered an added value with further improvements of daytime functions.

**Trial Registration:**

ClinicalTrials.gov Identifier: NCT01651442

## Key Points

**Question:**

Which first-stage treatment is optimal for improving daytime functions among patients with insomnia, and which second-stage treatment offers the best added value for patients whose insomnia has not remitted?

**Findings:**

In a randomized clinical trial of 211 adults with insomnia disorder, first-stage treatment with behavioral therapy (BT) or zolpidem produced significant improvements for various daytime outcomes, including depressive symptoms, fatigue, functional impairments, and mental health, that were no different between groups. Adding a second-stage therapy offered an added value for further improving daytime functions with immediate and delayed effects observed for treatment sequences starting with zolpidem and BT, respectively.

**Meaning:**

These findings support the comparable efficacy between sequential treatments starting with BT and zolpidem for addressing the daytime consequences of insomnia.

## Teaser

This randomized clinical trial compares the efficacy of behavioral therapy and zolpidem as initial therapies for improving daytime functions among patients with insomnia and evaluates the added value of a second treatment for patients whose insomnia has not remitted.

## Introduction

Insomnia is a highly prevalent sleep disorder that tends to be persistent or recurrent^1,2,3,4^ and can produce a significant burden on the individual and society.^5,6,7^ Insomnia is a 24-hour disorder that consists not only of nocturnal symptoms (ie, difficulty initiating and/or maintaining sleep) but also daytime symptoms.^8,9,10^ As one of the key components for insomnia diagnosis, daytime functional impairments such as fatigue and mood disturbances are often the primary reasons for patients with insomnia to seek treatment,^11,12^ indicating the necessity of addressing daytime consequences of insomnia.

Current recommended treatments for insomnia in clinical practice guidelines include 2 major approaches: psychological therapies (ie, cognitive behavioral therapy [CBT]) and medications (eg, benzodiazepine-receptor agonists and sedating antidepressants).^13,14,15^ Although medications are more frequently used in clinical practice mostly because they are more easily accessible, few data exist about the efficacy of sleep-promoting medications for improving daytime functions. With regard to CBT, the main outcomes are related to improving sleep continuity and insomnia severity, yet some evidence also suggests that it may be effective in improving various daytime symptoms, such as depressive and anxiety symptoms, daytime sleepiness, fatigue, and quality of life, among insomnia patients and those with comorbid depressive disorder.^16,17,18,19^ However, the effects of CBT on daytime symptoms are predominantly small to moderate, as revealed by a recent meta-analysis.^16^

Only a few studies have directly compared the efficacy of CBT and medications for daytime symptoms in insomnia, with mixed findings. In a randomized clinical trial (RCT) of young and middle-aged patients with chronic sleep-onset insomnia, no significant differences were found among CBT, medications, and combination therapy on mood-related changes after intervention.^20^ Another RCT of older patients with chronic insomnia found that CBT was more effective than zopiclone in reducing anxiety symptoms in the long term, and equally effective in reducing depressive symptoms in the short term.^21^ Furthermore, in an exploratory study of sequential treatments involving both medication and CBT, only CBT provided alone led to significant improvements in depressive symptoms from baseline to postintervention.^22^ These inconsistent results preclude definite conclusions about optimal therapy for improving daytime symptoms among patients with insomnia disorder.^23^ Although previous insomnia treatment studies have focused primarily on improving nocturnal symptoms of sleep continuity, greater attention to residual daytime impairments is important to optimize long-term outcomes. In addition, it is common for patients with insomnia who had suboptimal treatment responses to switch from one therapy to another in clinical practice. However, to our knowledge, no study has explored which first-stage treatment is optimal for improving daytime functions among patients with insomnia and which second-stage treatment offers the best added value for patients whose insomnia has not remitted with psychological or medication first-stage therapy.

The main objectives of this study were to compare short-term and long-term changes in daytime functions of 4 treatment sequences using psychological (behavioral and cognitive) and pharmacologic therapies (zolpidem and trazodone) for insomnia. In particular, the study aimed to compare the efficacy of behavioral therapy (BT) and zolpidem as first-stage therapies for improvement of daytime functions. For those whose insomnia did not remit after first-stage therapy, the added value of a second treatment was evaluated. Daytime functions investigated in the current study were among the predefined secondary outcomes of a previously published trial.^24^ Previous publications regarding the primary outcome related to sleep have suggested the benefits of sequential treatment paradigms on improving nocturnal symptoms of insomnia.^25,26^ We hypothesized that (1) participants receiving BT would show greater improvements in daytime functions after first-stage treatment than those receiving zolpidem and (2) of all patients receiving second-stage treatment, those who switched modalities (from BT to zolpidem, or vice versa) would report greater improvements at the end of second-stage treatment and at follow-ups than those continuing with the same treatment modality.

## Methods

### Study Design

This study is part of an RCT that aimed to examine the comparative efficacy of 4 treatment sequences involving psychological and medication therapies for insomnia with and without comorbid psychiatric disorder (trial protocol in Supplement 1). Details of the study design, methods, and primary results can be found elsewhere.^24,25,26^ The study was conducted at 2 sites: Institut Universitaire en Santé Mentale de Québec, Université Laval, Québec City, Québec, Canada, and National Jewish Health, Denver, Colorado. Participants were enrolled between May 1, 2012, and December 31, 2015. Ethical approval for the study was granted by the local ethics committees of both sites and all participants provided written informed consent. This study followed the Consolidated Standards of Reporting Trials (CONSORT) reporting guideline.

This project adopted a sequential multiple assignment randomized trial (SMART) design with 2 treatment stages and 2 treatment modalities for each stage (Figure).^25^ Eligible participants were randomly assigned to BT or zolpidem at a 1:1 ratio. The first randomization was conducted at each site and stratified by sex, age (<55 vs ≥55 years), and presence of a comorbid psychiatric disorder. After the initial 6 weeks of therapy, patients who met insomnia remission criteria were followed up for the next 12 months while receiving maintenance therapy. Participants were regarded as meeting insomnia remission criteria if their Insomnia Severity Index (ISI) score was less than 8 at the end of first treatment stage.^27^ Patients whose insomnia did not remit were randomized (stratified by first randomization and comorbidity) to a second-stage psychological therapy (BT or cognitive therapy [CT]) or medication therapy (zolpidem or trazodone). Measurements were conducted at baseline, end of first-stage therapy (post1) and second-stage therapy (post2), and at 3- and 12-month follow-ups. A complete description of the study protocol is available in Supplement 1.^24^

**Figure.**
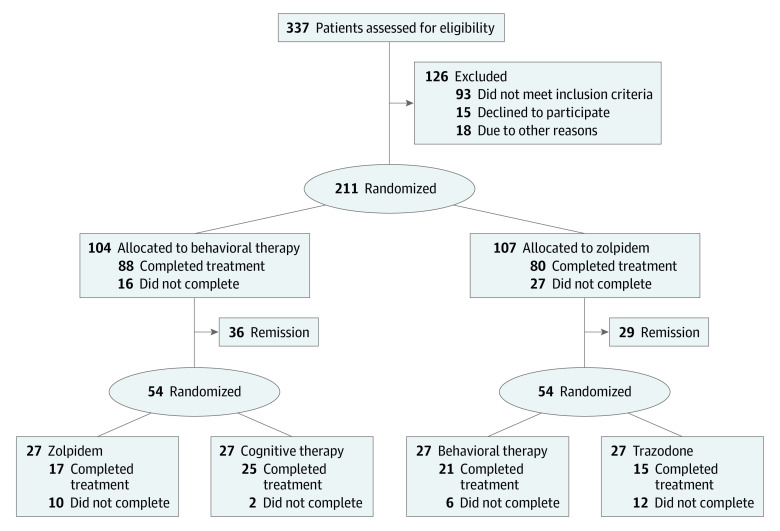
Participant Flow in the Sequential Treatment for Insomnia Reprinted with permission from Morin et al.^25^

### Participants

A total of 211 adults with chronic insomnia were recruited from the community through media advertisements and physician referrals. All participants included in the study met the following criteria: (1) aged 21 years or older, (2) persistent (>1 month) difficulties initiating or maintaining sleep despite adequate opportunity for sleep, (3) sleep onset latency or wake time after sleep onset of 30 minutes or more for 3 or more nights per week during 2 weeks of sleep diary monitoring, (4) ISI total score more than 10, (5) score of 2 or more on either the interference or distress item of the screening ISI. Participants were excluded if they had untreated psychiatric disorders or uncontrolled medical conditions or had conditions that interfered with sleep quality and sleep continuity. Additional details about exclusion criteria can be found in a previous publication.^25^ Self-reported race and ethnicity were collected to determine if the study population accurately represents the disease population.^28^

### Outcome Measures

The current study focused on changes in daytime functional outcomes, including mood disturbances, fatigue, functional impairments of insomnia, and the 36-item Short-Form Health Survey (SF-36) physical and mental health components.^29^ Changes in mood status were assessed with the Beck Depression Inventory–II (BDI-II)^30^ and the Trait part of the State-Trait Anxiety Inventory (STAI-Trait).^31^ Different dimensions of fatigue (eg, physical and mental) were measured by the Multidimensional Fatigue Inventory (MFI).^32^ The Work and Social Adjustment Scale (WSAS) was used to assesses the functional effect of insomnia on 5 domains: ability to work, home management, social leisure activities, private leisure activities, and relationships.^33^ Physical and mental health were evaluated by the SF-36,^29^ a quality-of-life measure, with a higher score representing a more favorable health state.

### Treatments

#### Cognitive Behavioral Therapy

The first-stage psychological therapy consisted of BT, which included sleep restriction^34^ and stimulus control procedures.^35^ The second-stage psychological treatment consisted of CT, which targeted some perpetuating mechanisms (eg, ruminations and worries) that are implicated in the association between insomnia and mood disturbances (eg, anxiety and depression).^36^

#### Medication

The first-stage medication treatment involved sublingual zolpidem, 5 mg to 10 mg, taken nightly at bedtime. The second-stage pharmacotherapy consisted of trazodone, 50 to 150 mg, taken 30 minutes before bedtime. As a serotonin receptor antagonist and reuptake inhibitor antidepressant, trazodone can alleviate a wide range of depressive symptoms with an additional sedative effect of sleep,^37^ and it has shown efficacy for both patients with insomnia and those with comorbid major depression.^38^ Additional information about treatment implementation can be found in a previous publication.^25^

### Statistical Analysis

The intention-to-treat analyses were performed in April and October 2023. To evaluate each treatment sequence while taking into account the nature of the SMART design (ie, 2 randomizations, where the second is conditional on the response to the first), the analytic strategy was based on recommendations from Nahum-Shani et al.^39^ Daytime functional outcomes according to 4 treatment sequences and 5 times (assessment after first-stage treatment [post1] to follow-up at 12 months) were analyzed using weighted generalized estimating equations models with identity link function.^40^ Weights were computed as the product of a missing model weight (to attenuate the effect of missing at random data) and a randomization weight (to correct for the inclusion of data from the patients whose insomnia remitted in stage 1 in the 2 sequences at stage 2 and beyond). Strata variables (site, age, sex, and comorbidity status) and outcome value at baseline were included as covariates.^41^ A priori contrasts within the weighted generalized estimating equations models were used to estimate temporal changes (baseline to post1, post1 to post2, and post2 to 12-month follow-up) and their 95% CIs and to test significance for comparisons between and within sequences. Data analyses were performed using the SAS, version 9.4, statistical software (SAS Institute Inc) with standard 2-tailed *P* < .05 considered statistically significant (*P* values for comparisons between sequences were adjusted for multiplicity using the simultaneous test procedure^42^).

## Results

### Participants

A total of 211 adults (132 women [63%]; mean [SD] age, 45.6 [14.9] years; 14 Black participants [7%], 11 Hispanic participants [5%], 182 White participants [86%], and 4 participants [2%] of other race or ethnicity [Asian (n = 2), Middle Eastern origin (n = 1), and not specified (n = 1)]) who met criteria for insomnia disorder (mean [SD] duration, 13.2 [12.5] years) were randomly allocated to BT (n = 104) or zolpidem treatment (n = 107) (Table 1).^25^ Seventy-four participants (35%) had a comorbid psychiatric disorder (eg, anxiety and depression). Use of psychotropic medication (other than sleep-promoting) was reported by 35 participants (17%) at baseline.

**Table 1.** Sociodemographic and Clinical Characteristics of Participants

Characteristic	Behavioral therapy (n = 104)	Zolpidem (n = 107)	Total sample (N = 211)
Study site, No. (%)			
Université Laval, Québec City, Québec, Canada	59 (57)	62 (58)	121 (57)
National Jewish Health, Denver, Colorado	45 (43)	45 (42)	90 (43)
Age, mean (SD), y	45.9 (14.4)	45.4 (15.5)	45.6 (14.9)
Sex, No. (%)			
Female	64 (62)	68 (64)	132 (63)
Male	40 (39)	39 (36)	79 (37)
Race and ethnicity, No. (%)			
Black	6 (6)	8 (8)	14 (7)
Hispanic	4 (4)	7 (7)	11 (5)
White	92 (88)	90 (84)	182 (86)
Other^a^	2 (2)	2 (2)	4 (2)
Educational level, mean (SD), y	16.3 (2.6)	16.0 (3.8)	16.1 (3.3)
Occupation, No. (%)			
Employed, full time	53 (52)^b^	57 (53)	110 (52)
Employed, part time	11 (11)	25 (23)	36 (17)
Student	6 (6)	4 (4)	10 (5)
Unemployed	13 (13)	4 (4)	17 (8)
Retired	20 (19)	17 (16)	37 (18)
Duration of insomnia, mean (SD), y	13.9 (12.3)	12.5 (12.6)	13.2 (12.5)
Psychiatric comorbidity, No. (%)	36 (35)	38 (36)	74 (35)
Medical comorbidity, No. (%)	66 (67)^b^	71 (69)^b^	137 (68)^b^
Use of sleep-promoting prescribed medication in the last year, No. (%)	25 (26)^b^	24 (23)^b^	49 (25)^b^
Use of psychotropic medication at baseline (other than sleep promoting), No. (%)	18 (18)^b^	17 (16)	35 (17)

Reprinted with permission from Morin et al.^25^

^a^ Asian (n = 2), Middle Eastern origin (n = 1), and not specified (n = 1).

^b^ Percentages do not total 100 due to missing data.

Of the 211 randomized participants, 168 completed first-stage therapy (Figure),^25^ including 88 in the BT group and 80 in the zolpidem group. There were no significant differences in attrition rates between the 2 groups (BT, 16 [15%] vs zolpidem, 27 [25%]; *P* = .15). After first-stage therapy, 36 participants in the BT group and 29 in the zolpidem group reached insomnia remission criteria (ISI score <8). Of the available participants whose insomnia did not remit, 108 accepted randomization to second-stage treatment (27 per condition). At baseline, the mean (SE) scores of the STAI-Trait and BDI-II for the included participants in both groups (STAI-Trait: BT group, 39.2 [0.4]; zolpidem group, 38.5 [0.4]; and BDI-II: BT group, 9.0 [0.4]; zolpidem group, 9.7 [0.4]) were below the cutoff scores for clinical mood impairments.

### Daytime Functional Outcomes

Table 2 shows adjusted mean (SE) values for psychological and daytime functional outcomes. Significant reduction in anxiety symptoms (STAI-Trait) was made with BT after first-stage treatment (mean change, −4.1 [95% CI, −5.8 to −2.4]; Cohen *d* = 0.79), but not with zolpidem therapy (−1.2 [95% CI, −3.0 to 0.5]; *d* = 0.24), and the difference between changes was significant (*P* = .02; *d* = 0.55) (eTable in Supplement 2). At post2, a further reduction of anxiety was observed in both conditions starting with zolpidem (mean change: zolpidem plus BT, −4.4 [95% CI, –7.5 to –1.2]; *d* = 0.85; zolpidem plus trazodone, −1.8 [95% CI, –3.4 to −0.3]; *d* = 0.36), while no change was observed in the sequences starting with BT. Comparisons for post1 to post2 changes of the 4 sequences at post2 was significant for STAI-Trait score (eTable in Supplement 2).

**Table 2.** Temporal Changes of Daytime Functional Outcomes

Stage 1 treatment	Pre score, mean (SE)^a^	Post1 score, mean (SE)	Score change from pre to post1, mean (SE) [95% CI]	Stage2 treatment	Post2, mean (SE)^b^	Score change from post1 to post2, mean (SE) [95% CI]	3-mo Follow-up score, mean (SE)	12-mo Follow-up score, mean (SE)	Score change from post2 to 12-mo follow-up, mean (SE) [95% CI]
**STAI-Trait (769 observations)**									
BT	39.2 (0.4); n = 104	35.2 (0.8); n = 84	−4.1 (0.9) [−5.8 to −2.4]^c^	BT + ZOL	36.0 (1.5); n = 23 (50)	0.5 (1.4) [−2.2 to 3.1]	32.8 (1.0); n = 20 (49)	31.4 (0.9); n = 20 (46)	−4.6 (1.6) [−7.7 to −1.5]^c^
				BT + CT	36.0 (0.9); n = 25 (52)	1.3 (0.8) [−0.4 to 2.9]	35.2 (1.2); n = 23 (52)	32.9 (1.1); n = 24 (50)	−3.1 (0.8) [−4.6 to −1.7]^c^
ZOL	38.5 (0.4); n = 107	37.2 (0.8); n = 79	−1.2 (0.9) [−3.0 to 0.5]	ZOL + BT	33.8 (1.6); n = 21 (44)	−4.4 (1.6) [−7.5 to −1.2]^c^	33.6 (1.9); n = 19 (42)	31.3 (2.1); n = 17 (39)	−2.5 (1.6) [−5.6 to 0.6]
				ZOL + TRAZ	34.5 (0.9); n = 19 (42)	−1.8 (0.8) [−3.4 to −0.3]^c^	34.0 (1.3); n = 18 (41)	32.8 (1.6); n = 16 (38)	−1.7 (1.4) [−4.4 to 0.9]
**BDI-II (769 observations)**									
BT	9.0 (0.4); n = 104	5.5 (0.4); n = 89	−3.5 (0.6) [−4.7 to −2.3]^c^	BT + ZOL	5.6 (1.0); n = 23 (52)	0.1 (0.8) [–1.5 to 1.8]	4.2 (0.6); n = 20 (49)	3.6 (0.5); n = 19 (45)	−2.0 (1.1) [−4.2 to 0.1]
				BT + CT	6.0 (0.7); n = 26 (55)	0.5 (0.6) [–0.8 to 1.7]	5.6 (0.7); n = 23 (52)	4.5 (0.7); n = 23 (49)	−1.5 (0.7) [–2.8 to −0.2]^c^
ZOL	9.7 (0.4); n = 107	5.4 (0.5); n = 81	−4.3 (0.7) [−5.7 to −2.9]^c^	ZOL + BT	3.0 (1.0); n = 21 (45)	−2.8 (1.1) [−4.9 to −0.6]^c^	3.4 (1.1); n = 19 (42)	1.0 (1.2); n = 17 (39)	−2.0 (1.5) [−5.0 to1.0]
				ZOL + TRAZ	4.6 (0.7); n = 20 (44)	−0.5 (0.5) [−1.5 to 0.6]	4.3 (0.9); n = 18 (41)	3.8 (0.9); n = 16 (38)	−0.7 (0.8) [−2.3 to 0.8]
**MFI (769 observations)**									
BT	52.6 (0.6); n = 104	47.9 (1.2); n = 84	−4.7 (1.3) [−7.3 to −2.2]^c^	BT + ZOL	45.8 (1.6); n = 23 (50)	−1.5 (1.7) [−4.7 to 1.8]	43.0 (1.9); n = 20 (49)	40.9 (1.6); n = 20 (46)	−4.9 (1.5) [−7.9 to −1.9]^c^
				BT + CT	46.1 (1.9); n = 25 (52)	−2.5 (1.8) [−6.0 to 1.0]	44.6 (1.7); n = 23 (52)	42.0 (1.9); n = 24 (50)	−4.1 (1.5) [−6.9 to −1.2]^c^
ZOL	52.2 (0.6); n = 107	47.0 (1.3); n = 79	−5.2 (1.4) [−7.9 to −2.5]^c^	ZOL + BT	42.2 (1.8); n = 21 (44)	−3.8 (1.7) [−7.1 to −0.4]^c^	42.1 (2.6); n = 19 (42)	40.9 (2.5); n = 17 (39)	−1.3 (1.9) [−5.1 to 2.5]
				ZOL + TRAZ	44.3 (1.7); n = 19 (42)	−3.7 (1.3) [–6.3 to −1.1]^c^	44.4 (2.2); n = 18 (41)	41.7 (2.7); n = 16 (38)	−2.5 (2.2) [−6.9 to 1.8]
**WSAS (767 observations)**									
BT	16.1 (0.6); n = 104	11.1 (0.8); n = 83	−5.0 (0.9) [−6.7 to −3.3]^c^	BT + ZOL	10.3 (1.4); n = 23 (50)	−1.0 (1.4) [−3.7 to 1.7]	9.1 (1.1); n = 20 (49)	7.6 (1.3) n = 20 (46)	−2.8 (1.3) [−5.3 to −0.2]^c^
				BT + CT	8.3 (1.0); n = 25 (52)	−2.6 (0.9) [−4.4 to −0.7]^c^	7.0 (1.3); n = 23 (52)	6.1 (1.1) n = 24 (50)	−2.2 (0.9) [−3.9 to −0.5]^c^
ZOL	16.8 (0.6); n = 107	11.7 (1.0); n = 79	−5.1 (1.1) [−7.2 to −2.9]^c^	ZOL + BT	7.1 (1.0); n = 21 (44)	−3.7 (1.4) [−6.4 to −1.0]^c^	7.1 (1.8); n = 19 (42)	7.5 (1.7); n = 17 (38)	0.4 (1.1) [−1.7 to 2.5]
				ZOL + TRAZ	9.4 (1.4); n = 19 (42)	−3.3 (1.3) [−5.9 to −0.7]^c^	6.8 (1.4); n = 18 (41)	9.1 (1.8); n = 16 (37)	−0.2 (1.4) [−2.9 to 2.4]
**SF-36 Physical Health subscale (767 observations)**									
BT	49.0 (0.3); n = 104	49.1 (0.7); n = 83	0.1 (0.8) [−1.4 to 1.7]	BT + ZOL	48.2 (1.2); n = 23 (50)	−0.4 (1.2) [−2.7 to 2.0]	47.7 (1.2); n = 20 (49)	50.7 (0.9); n = 20 (46)	2.5 (1.3) [−0.2 to 5.1]
				BT + CT	49.7 (1.2); n = 25 (52)	0.1 (1.2) [−2.2 to 2.4]	50.4 (1.1); n = 23 (52)	51.6 (1.1); n = 23 (49)	1.9 (1.3) [−0.6 to 4.4]
ZOL	47.7 (0.5); n = 107	49.5 (0.7); n = 79	1.8 (0.9) [0.1 to 3.6]^c^	ZOL + BT	49.8 (0.9); n = 21 (44)	−0.3 (0.9) [−2.0 to 1.4]	49.7 (1.4); n = 19 (42)	50.0 (1.3); n = 17 (39)	0.3 (0.9) [−1.4 to 2.0]
				ZOL + TRAZ	48.5 (1.0); n = 19 (42)	−0.3 (1.0) [−2.2 to 1.5]	48.3 (1.1); n = 18 (41)	49.6 (1.0); n = 16 (38)	1.1 (1.1) [−1.1 to 3.3]
**SF-36 Mental Health subscale (767 observations)**									
BT	46.5 (0.5); n = 104	49.9 (0.8); n = 83	3.5 (0.8) [1.9 to 5.1]^c^	BT + ZOL	48.9 (1.1); n = 23 (50)	−1.3 (1.2) [−3.7 to 1.2]	50.7 (1.0); n = 20 (49)	50.1 (1.0); n = 20 (46)	1.3 (1.2) [−1.2 to 3.7]
				BT + CT	48.7 (1.4); n = 25 (52)	−1.0 (1.4) [−3.7 to 1.7]	50.1 (1.0); n = 23 (52)	50.3 (1.3); n = 23 (49)	1.6 (1.2) [−0.7 to 3.9]
ZOL	46.7 (0.5); n = 107	49.2 (0.9); n = 79	2.5 (1.0) [0.4 to 4.5]^c^	ZOL + BT	54.1 (1.3); n = 21 (44)	5.3 (1.3) [2.7 to 7.9]^c^	53.3 (1.4); n = 19 (42)	53.8 (1.2); n = 17 (39)	−0.2 (1.3) [−2.7 to 2.2]
				ZOL + TRAZ	51.6 (1.2); n = 19 (42)	2.0 (1.0) [0.1 to 4.0]^c^	53.2 (1.5); n = 18 (41)	52.5 (1.6) ; n = 16 (38)	0.9 (1.5) [−2.1 to 3.9]

Abbreviations: BDI-II, Beck Depression Inventory-II; BT, behavioral therapy; CT, cognitive therapy; MFI, Multidimensional Fatigue Inventory; pre, before stage 1 of treatment; post1, after stage 1 of treatment; post2, after stage 2 of treatment; SF-36, 36-item Short-Form Health Survey; STAI-Trait, State-Trait Anxiety Inventory; TRAZ, trazodone; WSAS, Work and Social Adjustment Scale; ZOL, zolpidem.

^a^ All mean values are adjusted for outcome value at baseline, age, sex, site, and psychiatric comorbidity covariates.

^b^ For post2, 3-month follow-up, and 12-month follow-up time points, the number of patients randomized to each stage2 treatment is indicated in each cell, but as analyses were weighted using a randomization weighting (ie, patients whose insomnia remitted at stage 1—not being randomized at stage 2—are included in both possible stage 2 sequences), the total number of observations used to estimate each cell mean and SE is also reported in parentheses.

^c^ *P* < .05.

Both first-stage therapies produced significant improvements for depressive symptoms (mean change in BDI-II score: BT, −3.5 [95% CI, −4.7 to −2.3]; *d* = 0.90; zolpidem, −4.3 [95% CI, −5.7 to −2.9]; *d* = 1.10), fatigue (mean change in MFI score: BT, −4.7 [−7.3 to −2.2]; *d* = 0.47; zolpidem, −5.2 [95% CI, −7.9 to −2.5]; *d* = 0.64), functional impairments (mean change in WSAS score: BT, −5.0 [95% CI, −6.7 to −3.3]; *d* = 0.78; zolpidem, −5.1 [95% CI, −7.2 to −2.9]; *d* = 0.80), and the SF-36 mental health subscale (mean change: BT, 3.5 [95% CI, 1.9-5.1]; *d* = 0.52; zolpidem, 2.5 [95% CI, 0.4-4.5]; d = 0.38); significant improvements for the SF-36 physical health subscale were made only with zolpidem as first-stage treatment (mean change, 1.8 [95% CI, 0.1-3.6]; *d* = 0.35; Table 2). There were no significant group differences for any of those measures (eTable in Supplement 2).

At post2, further improvements were observed for fatigue (mean MFI score change: zolpidem plus BT, −3.8 [95% CI, −7.1 to −0.4]; *d* = 0.36; zolpidem plus trazodone, −3.7 [95% CI, −6.3 to −1.1]; *d* = 0.46), functional impairments (mean WSAS score change: zolpidem plus BT, −3.7 [95% CI, −6.4 to −1.0]; *d* = 0.62; zolpidem plus trazodone, −3.3 [95% CI, −5.9 to −0.7]; *d* = 0.55) and the SF-36 mental health subscale (mean score change: zolpidem plus BT, 5.3 [95% CI, 2.7-7.9]; *d* = 0.91; zolpidem plus trazodone, 2.0 [95% CI, 0.1-4.0]; *d* = 0.35) in treatment sequences starting with zolpidem, while for depression symptoms, only the zolpidem plus BT sequence showed significant improvements (mean BDI-II score change, −2.8 [95% CI, −4.9 to −0.6]; *d* = 0.71) (Table 2). No additional changes were observed in the 2 sequences starting with BT at post2, except for functional impairments (mean WSAS score change: BT plus CT, −2.6 [95% CI, −4.4 to −0.7]; *d* = 43). The comparisons of the 4 sequences yielded a significant overall effect only for the SF-36 mental health subscale at post 2 (eTable in Supplement 2), with both conditions starting with zolpidem showing higher scores on the SF-36 mental health subscale.

### Follow-Up Assessments

Both conditions starting with BT showed significant improvements from post2 to 12-month follow-up for anxiety symptoms (mean change in STAI-Trait score: BT plus zolpidem, −4.6 [95% CI, −7.7 to −1.5]; *d* = 0.90; BT plus CT, −3.1 [95% CI, −4.6 to −1.7]; *d* = 0.61), fatigue (mean change in MFI score: BT plus zolpidem, −4.9 [95% CI, −7.9 to −1.9]; *d* = 0.60; BT plus CT, −4.1 [95% CI, −6.9 to −1.2]; *d* = 0.50), and functional impairments (mean change in WSAS score: BT plus zolpidem, −2.8 [95% CI, −5.3 to −0.2]; *d* = 0.46; BT plus CT, −2.2 [95% CI, −3.9 to −0.5]; *d* = 0.37) (Table 2). For depressive symptoms, only the BT plus CT sequence showed a significant improvement from post2 to 12-month follow-up (mean change in BDI-II score: −1.5 [95% CI, −2.8 to –0.2]; *d* = 0.39). No group differences were obtained at follow-ups for any of the outcomes (eTable in Supplement 2).

### Subgroup Analysis for Participants With Psychiatric Comorbidity

The findings in the subgroup analysis by psychiatric comorbidity (absent vs present) were similar to those from the main analysis for most of the outcomes. However, they are not reported in detail here due to the small sample sizes and reduced power for those analyses.

## Discussion

The current study showed that first-stage treatment with BT or zolpidem was effective in reducing daytime symptoms of insomnia, with no significant differences between groups. The addition of a second-stage therapy resulted in an added value in enhancing daytime functions. In particular, immediate effects of second-stage therapies were observed for sequences that used zolpidem as the initial treatment, while delayed effects were made with sequences starting with BT at 12-month follow-up. Overall, these findings provide further support for the efficacy of CBT and sleep-promoting medication for improving daytime functions among patients with insomnia.

Although some studies have previously reported benefits of CBT on daytime outcomes,^16,17,18,19^ reports of daytime functional improvements with medication therapy are relatively novel, to our knowledge.^43^ A significant reduction in anxiety symptoms was seen with BT after first-stage treatment, but not with zolpidem, which is similar to the findings of a previous study conducted among patients with insomnia without psychiatric comorbidity.^21^ The relative superiority of BT compared with zolpidem for reducing anxiety symptoms may be partly explained by the significant reduction in sleep latency and time awake after sleep onset made with BT, which could lead to reduced anxiety and worry when initiating sleep and during midnight awakenings.^25^ In addition, BT may also reduce sleep-related anxiety or distress through modifying participants’ perception of sleep.^44^ However, this finding should be interpreted with caution, as the threshold for clinically significant anxiolysis should be a 50% or greater reduction in baseline total score.^45,46^

At the end of second-stage therapy, although patients in the 2 groups starting with zolpidem showed further improvements for most daytime outcomes, only patients who switched from pharmacotherapy to psychological treatment (from zolpidem to BT) reported greater reductions in depressive severity. This finding aligned with our hypothesis that switching treatment modalities would lead to greater treatment benefits, although it needs to be interpreted cautiously given the smaller sample size for the treatment sequence involving 2 medications and the mild range of depressive symptoms. In the treatment sequences starting with BT, patients who received 2 treatments within the same modality (from BT to CT) had slightly better outcomes than those who switched modality (from BT to zolpidem) at 12-month follow-up, which may be partially attributed to the broader action of CT by targeting psychological and mood symptoms compared with zolpidem.^36,47^

### Limitations

Some of the findings need to be interpreted cautiously given some methodological limitations. First, the lack of a control condition and the relatively small sample sizes for each treatment sequence may reduce the statistical power to detect more significant group differences. Second, only patients whose insomnia did not remit received second-stage therapy, while patients with insomnia who achieve remission can still have residual daytime impairments (eg, fatigue and mood disturbances) that are associated with future relapse^48^ and may be adequately addressed only by maintenance therapy. Additional studies should consider including daytime outcomes as one of the measures to guide treatment decisions. Third, the study population may not accurately represent the target disease population due to its underrepresentation of racial and ethnic minority groups,^28^ which could potentially affect the generalizability of the findings to the broader population of individuals.

## Conclusions

The present study documented the efficacy of BT and zolpidem for improving daytime functional outcomes among patients with insomnia and the effect of BT on reducing anxiety symptoms. Adding a second treatment offered an added value for further improvements of daytime functions. Future developments of insomnia treatment strategies should take into account the daytime consequences of insomnia. Additional studies are needed to further investigate the potential benefits of switching treatment modalities and incorporating a therapeutic component that can address psychological and mood disturbances.

## Notes

Supplement 1.
Trial Protocol

Supplement 2. **eTable.** Significance Level of Between-Sequence Statistical Comparisons

Supplement 3.
Data Sharing Statement
