# Supplementary material for: Effect of Psychological and Medication Therapies for Insomnia on Daytime Functions: A Randomized Clinical Trial
Source: JAMA Netw Open. 2023 Dec 28;6(12):e2349638. doi: 10.1001/jamanetworkopen.2023.49638 (PMC10755607; doi:10.1001/jamanetworkopen.2023.49638)
Supplement: Supplement 1. — Trial Protocol [file jamanetwopen-e2349638-s001.pdf]

3. RESEARCH STRATEGY*

(a) Significance: Insomnia is characterized by difficulties initiating, sustaining, or obtaining qualitatively satisfying sleep that occur despite adequate sleep opportunities/circumstances and result in notable waking deficits[3, 4]. Over 33% of adults experience insomnia at least intermittently, whereas 10% to 15% suffer chronic, unrelenting sleep difficulties[5-9]. Although its significance is often minimized[10, 11], persistent insomnia may lead to daytime fatigue, decreased mood, impairment in social/vocational functioning and reduced quality of life[12-15]. Insomnia also increases the risks for serious medical disorders, traffic and work-site accidents, alcohol/drug abuse, and major psychiatric illnesses[6, 16-22]. When insomnia occurs co-morbid to a psychiatric illness such as major depression, it complicates disease management and often remains as a residual symptom that enhances risk for both suicide and relapse[23-26]. Moreover, insomnia contributes to increased health care utilization and costs. Indeed, insomnia sufferers spend well over $285 million per year for prescription sleeping pills whereas the annual direct, treatment-related costs of insomnia in the U.S. may exceed $90 billion[27, 28]. More than 90% of insomnia-related costs are attributable to work absences and reduced productivity[29]. In view of these considerations, ascertaining the most effective and enduring treatments for the many who suffer chronic insomnia should be a priority for our health care system and for our nation.

WHAT INSOMNIA TREATMENT OPTIONS ARE CURRENTLY AVAILABLE? Several pharmacological treatment options are available for insomnia management. The most popular agents are the benzodiazepine receptor agonists (BzRAs) which bind to the $GABA_A$ receptor complex [28, 30]. In so doing, these agents enhance the sleep promoting effects of homeostatic sleep-drive and decrease activity in arousal systems (acetylcholine, histamine, orexin/hypocretin, serotonin, etc.)[31]. BZRAs include several benzodiazepines (e.g., temazepam, triazolam, etc.) and newer non-benzodiazepine agents (e.g., zolpidem, eszopiclone,). Additionally, the melatonin agonist, ramelteon, is FDA-approved for insomnia therapy, though its sleep improving mechanisms are unknown. Finally, off-label use of antihistamines and sedating anti-depressants such as trazodone (TRZ)[28] and tricyclic antidepressants (e.g., amitriptyline, doxepin)[28] has been popular for insomnia management. These agents enhance sleep by diminishing arousal through blocking the effects of wake-promoting systems[31].

Complementing these pharmacotherapies are a variety of psychological therapies. These treatments address one or more of the psychological/behavioral mechanisms thought to perpetuate insomnia such as maladaptive sleep habits, dysfunctional beliefs about sleep, excessive cognitive or physiological arousal, and poor sleep hygiene practices [32-35]. Included among these approaches are various stand-alone strategies such as stimulus control, sleep restriction, relaxation training, paradoxical intention, sleep hygiene education, and cognitive therapy. In addition, multi-component, cognitive-behavioral insomnia therapies (CBTs) that combine several of these therapies to optimize outcomes have become the most frequently used approaches.

HOW EFFECTIVE ARE THE AVAILABLE INSOMNIA THERAPIES? Of the various prescription medications used for insomnia treatment, FDA-approved BzRAs have the most efficacy and safety data and consequently have come to be regarded as first-line insomnia therapies. A meta analysis[36] of 22 placebo-controlled trials involving traditional BzRAs and zolpidem with primary insomnia (PI) patients showed these agents produce reliable short-term (median treatment duration = 7 days; range = 4-35 days) improvements of sleep-onset latency (mean effect size = 0.56), number of awakenings (effect size = 0.65), total sleep time (effect size = 0.71), and sleep quality (effect size = 0.62). Furthermore, a few recently published trials[37, 38] have shown the newer BzRAs such as zolpidem have continued efficacy and safety for periods of 3-12 months of nightly use. Whereas studies of BzRAs with insomnia occurring comorbid to a mental disorder (CMI) have been limited, there are some data showing that combining a BzRA with antidepressant medications (SSRIs) is significantly more efficacious than SSRIs alone for treating both insomnia and depression in patients with major depressive disorder[39-42]. In contrast, trazodone and most sedating tricyclic antidepressants lack FDA approval for insomnia management since data supporting their efficacy with insomnia patients is extremely limited. Yet, trazodone has been used widely "off-label" for insomnia treatment[28] and there are some data supporting the efficacy of this agent for treating insomnia occurring with ongoing mood disorders such as major depression[43-47].

Like the BzRAs the psychological/behavioral insomnia therapies are well supported and can be considered first-line treatments. Several meta-analyses[48-51] that considered over 50 published trials with PI sufferers show such treatments produce moderate to large improvements in sleep onset latency (effect sizes = .87-.88), total sleep time (effect sizes = 0.42-0.49), number of awakenings (effect sizes = 0.53-0.63), duration of awakenings (effect size = 0.65), and sleep quality ratings (effect size = 0.94). Overall, between 70%-80% of treated PI patients benefit from treatment, with the best outcomes resulting from multi-faceted therapies such as CBTs [33, 51-53]. Applications of these therapies to CMI patients have been more limited, but the available data suggest these treatments produce sleep improvements among insomnia patients with chronic peripheral pain

syndromes[54], breast cancer[55], fibromyalgia[56], mixed medical disorders[57], alcoholism[58], and depression [59, 60]. Previous findings also suggest that these therapies lead to improvements in mood status, enhanced likelihood of depression remission, and reductions in other disease-specific symptoms among CMI patients[55, 56, 59, 61, 62]. Thus, the psychological/behavioral therapies hold promise for CMI patients as well as for those with PI.

WHAT SHOULD BE OUR FIRST STAGE INSOMNIA THERAPY AND WHAT SHOULD WE DO WHEN THAT FAILS? Deciding whether to use pharmacological or psychological/behavioral insomnia therapy in general or with specific patients is difficult, since both forms of treatment have their limitations. Medications usually produce rapid improvements, are widely available, and generally well tolerated, but adverse effects including residual daytime sedation, reduced motor coordination, cognitive impairment, tolerance, and rebound effects may complicate their use[63-65],[66-68]. Furthermore, there are no data documenting enduring benefits of these agents after their use is discontinued. In contrast, the psychological/behavioral insomnia therapies have minimal side effects, are preferred by many patients, and typically result in enduring sleep improvements long after termination of acute treatment[48, 51]. However, these therapies require more extensive provider contact and have a slower rate of therapeutic action than do medications[69, 70]. In addition, they are less widely available than BzRAs despite recent efforts to provide their wider dissemination through abbreviated therapy protocols[71, 72], self-help interventions[73], and the training of non-traditional providers[74].

Whereas the relative value of BzRA and psychological/behavioral therapies largely depends upon their comparative efficacies and safeties, there have been few head-to-head comparisons of these treatments. One recent trial[75], which compared CBT with the BzRA, zopiclone, over a 6-week acute treatment phase and subsequent six-month follow-up, showed CBT produced significantly better short- and longer-term improvements on objective (PSG) indices but not on subjective (sleep diary) measures. A few other studies[69, 76, 77] that compared a single-agent BzRA therapy, CBT, and combined BzRA/CBT therapy showed little difference in short-term outcomes, but superior longer-term outcomes with CBT compared to BzRA and combined treatment. In contrast, a recently published trial showed a sequential treatment strategy that commenced with 6 weeks of combined CBT/BzRA therapy followed by an extended six months of CBT alone proved superior to continued long-term combined therapy or CBT provided in the absence of any medication [2]. However, these studies are limited by their small sample sizes, use of fixed-dose/fixed-agent pharmacotherapy strategies that do not represent standard clinical practice, and/or their exclusive focus on PI patients. Hence, these findings provide very limited guidance for deciding upon the optimal first-stage insomnia therapy in general and specifically for the prominent and challenging subgroup of CMI patients with psychiatric illnesses.

Further complicating matters are a number of additional oversights in the insomnia treatment literature that limit its usefulness for guiding clinical practice. First, most previous treatment studies have focused on changes in individual quantitative sleep measures (e.g. sleep onset latency, total sleep time) to gauge treatment outcomes. These indices are important, but they miss other relevant symptoms such as daytime fatigue, cognitive efficiency, and overall sleep satisfaction which patients regard as particularly important and clinicians use to assess global improvement[78-81]. Moreover, few studies have assessed treatments using validated measures of insomnia remission, the outcome most relevant to clinical practice. Secondly, it remains unclear how well CMI patients respond to the standard doses/protocols of insomnia therapies established for PI patients. Emerging evidence[82] suggests CMI patients demonstrate greater adherence difficulties to CBT than do PI patients, and our own data (see preliminary studies) indicate CMI patients show a less robust CBT response than do PI sufferers when global insomnia syndrome measures are considered. Likewise, recent studies[83] using the Insomnia Severity Index[84] to assess the efficacy of the BzRA, eszopiclone, show large treatment effect sizes = 0.87 for PI patients but smaller effect sizes = 0.46 and 0.48 in patients with comorbid generalized anxiety disorders and major depression respectively. CMI patients, thus, may have a more blunted response to first-stage insomnia therapy than do PI patients regardless of the specific modality they receive. Finally, extant data shows a large proportion of patients fail to remit with first-stage therapy and are, thus, left with residual insomnia symptoms.[51] In such situations, switching from one therapy to another, on a trial-error basis, is common clinical practice. Yet, no studies have addressed such important questions as: (1) *Which second-stage treatment offers the best hope for insomnia remission, once a reasonable course of a psychological or medication first-stage therapy fails to achieve this endpoint? and (2) Do the optimal first-stage-to-second-stage treatment sequences differ for those with CMI and PI?*

When selecting first stage therapies to test, we chose two distinctive treatment modalities that are well supported, relatively easy to administer, and frequently used. One is the medication, zolpidem, which has well established efficacy and is now the most often prescribed BzRA for insomnia[43]. Our alternate first line therapy will be a behavioral therapy (BT) comprised of stimulus control, sleep restriction, and sleep hygiene education.

Although multi-component CBT may represent the psychological treatment of choice, we have chosen to employ only its core behavioral elements so as to make this therapy easy to deliver and ultimately more transferable to clinical settings. BT and Zolpidem will also serve as second-stage therapies so that we can assess effects of switching from one type of sleep-focused therapy to another (e.g., BzRA→BT) We chose trazodone as an alternate second-stage pharmacotherapy since this medication has enjoyed wide off-label use as a sleep aid over the past several decades, a different mechanism of action than BzRAs, and proven efficacious among CMI patients with mood disorders[43, 46, 47]. Finally we chose to test the Cognitive Therapy (CT) component of CBT as an independent second-stage treatment. CT seems reasonable to choose as a second stage therapy since it is time consuming to deliver and not essential for all insomnia patients. Yet, CT is designed to target critical cognitive perpetuating mechanisms for insomnia (worries, self-monitoring, negative automatic thoughts), some of which may be shared with other psychiatric disorders (e.g., anxiety and depression). These cognitive factors are not specifically addressed by BT. Thus, CT's impact could be broader and improve mood and sleep, a very desirable outcome among patients with comorbid psychiatric disorders.

(b) Innovation: The proposed project entails a dual-site, open-label trial examining sequential treatment with well-supported and commonly used therapies to asses their relative efficacy and safety. This project has the following innovative features designed to advance understanding of the treatment needs of insomnia sufferers, particularly those with treatment-resistant psychiatric illnesses: (1) Enrollment of participants with broadly-defined chronic insomnia disorder, with and without psychiatric comorbidity; (2) use of the clinically-relevant primary outcome, insomnia remission, rather than the traditional, less relevant quantitative parameters (e.g., sleep time, sleep onset latency); (3) use of a sequential treatment design that tests various first-stage-to-second-stage treatment sequences; (4) flexible medication dosing, rather than a fixed-agent/fixed dose design; and (5) plans to systematically collect AE data for both psychological and medication therapies so that the relative safety of the two approaches can be examined. This project should provide new and relevant information that contributes to the development of clinical guidelines for CMI and PI management, guidelines which are critically lacking[85].

(c) Approach:

c.1 Study Design and Rationale. Adults with chronic insomnia (n = 320) will be randomly assigned to zolpidem therapy (MED; n = 160) or behavioral therapy (BT; n = 160), stratified by gender, age (< 55 years vs. ≥ 55 years), and insomnia subtype (primary-PI vs. comorbid-CMI). We expect roughly 50% of our sample will be ≥ 55 years old, about 60% will be women, and we will recruit 60% with insomnia comorbid to a mental disorder. We will monitor other variables (e.g., prior usage of hypnotic medications) that might be related to treatment response and, if necessary, control for these in statistical analyses. After completing the initial 6-week treatment phase, treatment remitters will remain on maintenance therapy. Non-remitters will be encouraged to accept randomization to a second-stage alternate therapy provided over the next 6 weeks. Participants treated with BT initially will receive another psychological treatment, CT, or a medication therapy (zolpidem). Those treated with medication (zolpidem) initially will be switched to BT or to a different medication (trazodone). Measurements will be taken at baseline, at the end of first- and second-stage therapies (i.e., weeks 6 and 12), and at follow-ups conducted 3, 6, 9, and 12 months after the week 12 assessment.

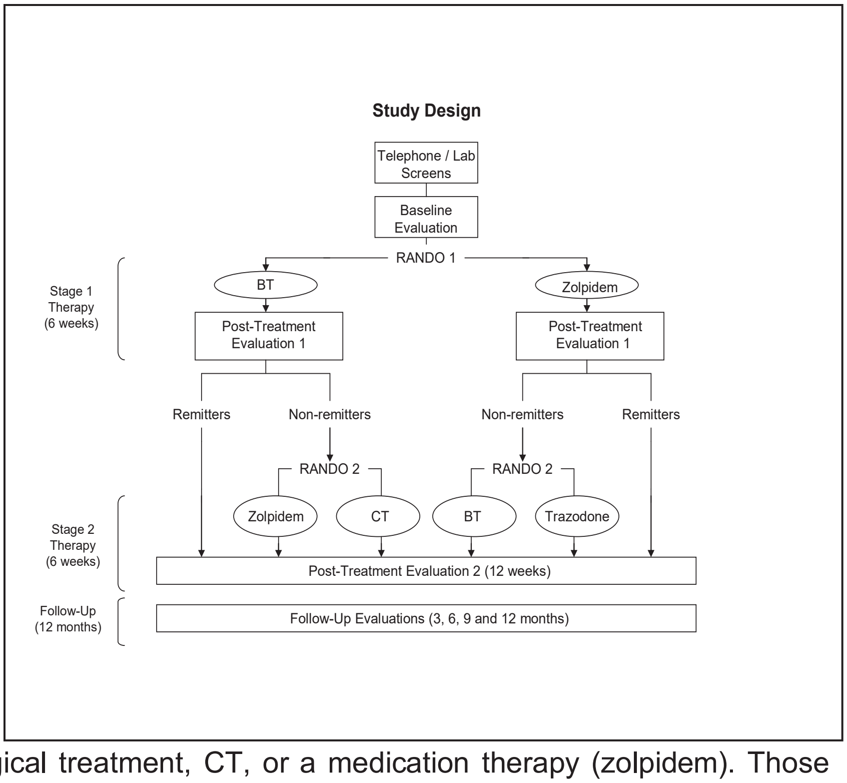

This study design derives from our primary aims: (1) to compare outcomes of BT and zolpidem when provided as first-stage therapies for insomnia patients with/without psychiatric comorbidity and (2) to assess the *value-added* of switching strategies for those not achieving remission with first-stage therapy. The BzRA, zolpidem is included because it is readily available and the most widely used pharmacotherapy for insomnia. BT was chosen for comparison because it is an effective, pragmatic, and safe treatment option that is preferred

by many patients. Evidence suggests both BZRAs and BT produce a treatment response in roughly 60%-70% of patients, although only about 40% of all patients achieve remission. The second-stage treatments will show whether added efforts involving changing therapy within a broad treatment class (psychological or medication), or switching to an alternate mode of therapy will bring more patients into remission.

Several alternative conditions were considered when planning this study. For instance, although CBT is becoming the standard approach to treating insomnia, we decided to introduce its two main therapeutic components (BT and CT) in a sequential fashion rather than as a treatment package. First, there is much stronger evidence supporting BT than CT; in addition, BT is easier to implement than CT (by non-specialists), thus enhancing its transferability to clinical practice. We also considered using a combined treatment involving concurrent use of medication and CBT but decided against this option because it is rarely available as a first-stage therapy and because the evidence suggest that combined CBT/MED therapy is not necessarily optimal, when long-term outcomes are considered. Our study design provides a unique opportunity to test the impact of sequential rather than concurrent therapies. We also considered including a placebo-control condition but decided not to do so because our research questions are not about documenting treatment efficacy relative to placebo; such data are already available. Rather, our main questions concern the comparative efficacy of first-stage psychological and pharmacological therapies and their optimal sequencing as second-stage therapies for CMI and PI. In the end, we believe the design chosen is the most appropriate to answer our specific research questions, while offering the best compromise in terms of feasibility, cost, and statistical power.

c.2 Preliminary Studies. The preliminary studies (a) show the PIs' experience and leadership in conducting RCTs of insomnia therapies, (b) provide rationale for the therapies chosen and study hypotheses and, (c) support the feasibility of the project proposed (See Appendix 1 for overall collaborative plan).

CBT and Medication for Persistent Insomnia: Short-Term and Maintenance Treatment Effects.[2] Dr. Morin's group conducted this study to evaluate the short-term effects of CBT, alone and combined with medication, and to compare effects of varied treatment sequencing strategies on long-term outcomes. A total of 160 chronic insomnia patients (61% women; mean age of 50.3 yrs) were randomized to CBT (n = 80) or CBT plus nightly 10 mg zolpidem (n = 80) for an initial 6-week treatment. After completing this treatment, they were randomized again for an extended 6-month treatment. Patients initially treated with CBT alone continued with extended individualized CBT or received no additional treatment; those who received combined treatment initially continued with an extended treatment consisting of CBT plus intermittent medication (10 pills per month) or CBT alone. Of the 160 patients enrolled, 148 completed acute treatment, 141 completed extended treatment, and 127 patients were available at 6-month and 125 at 12-month FUs (overall attrition rate of 22% over a 1-year period). Outcomes were examined in terms of treatment response (change on the Insomnia Severity Index (ISI) > 7) and remission (ISI value < 8). Although the proportion of treatment responders was comparable with CBT alone (60%) and CBT plus medication (61%) at the end of acute treatment, different trajectories of change emerged over the following 6 months depending on whether therapy was provided. More patients responded with maintenance CBT than without (63% vs. 55%). Also, patients treated with combined

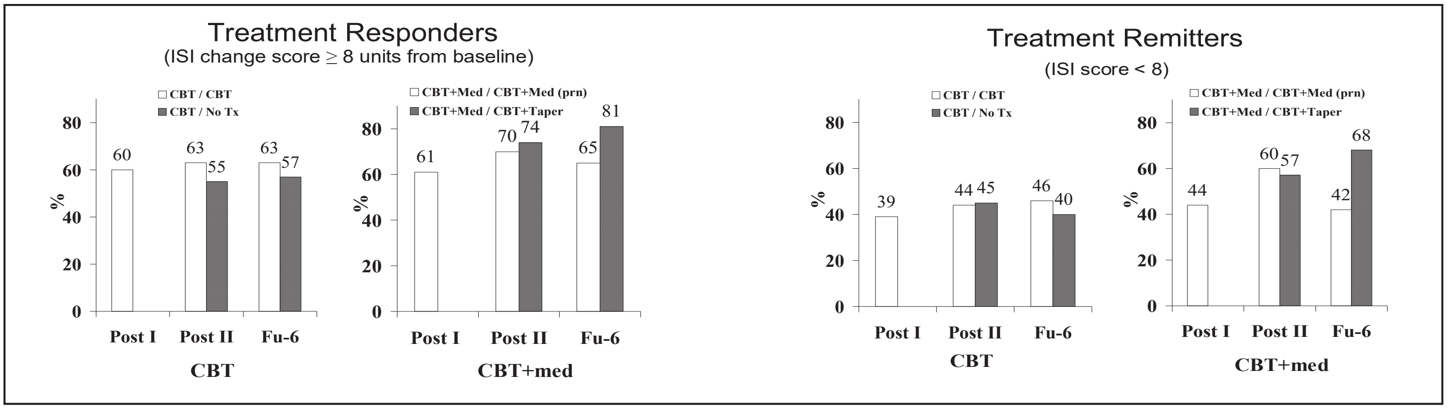

therapy initially did slightly better during maintenance therapy when medication was stopped compared to intermittent usage (74% vs. 70% responders), and this trend became significant at the 6-month follow-up (81% vs. 65%). A similar pattern emerged regarding remission. Remission rates were 39% (CBT alone) and 44% (CBT + medication) after acute therapy, and there was no significant difference after extended therapy between CBT and no additional treatment or between CBT alone and CBT plus intermittent medication. At the 6-month follow up, however, remission rates were significantly higher among those who had received maintenance CBT than among those who did not (46% vs. 40%) and among those who discontinued

medication versus those who continued with intermittent medication (68% vs. 42%). These findings, while preliminary, have implications for the current proposal. First, CBT + medication may not provide a strong advantage over CBT alone, at least on acute outcome. Second, the addition of a second-stage alternate psychological therapy may enhance long-term remission rates. Thirdly, when combined therapy is used as first-stage therapy, long-term remission rates may be enhanced by tapering medication while patients are still receiving CBT. Although informative, these conclusions remain tentative because the findings were based on a limited sample obtained at only one site and only patients with primary insomnia were enrolled. In addition, the study made no comparison between CBT alone and medication alone. Moreover, the randomization to second-stage therapy did not consider initial treatment response, a factor that should guide second-stage therapy.

Instability of sleep and bedtime arousal in CMI and PI patients[86]. This study conducted by Dr. Edinger's group, examined patterns of sleep disturbance and bedtime arousal in individuals with PI and CMI related to a mental disorder. The study included 187 insomnia sufferers (126 women, M_{Age} = 47 years) assigned either PI (n = 126) or CMI (n = 61) diagnoses by 6 sleep specialists at one or the other of two collaborating medical centers. Results showed CMI patients displayed significantly longer SOL, on average, and significantly more instability across nights in their TST (i.e., larger changes) than did PI patients. CMI patients exhibited higher levels of somatic, cognitive and emotional arousal as well as more instability on nightly ratings of emotional arousal. Correlations revealed a significant relationship between pre-sleep arousal and SOL in the PI group (r values from 0.26 to 0.34), whereas corresponding correlations between all sleep and bedtime arousal measures were non significant in the CMI group (r values < 0.14). Despite greater levels of arousal and longer SOLs in the CMI group, they showed less correspondence between sleep and arousal than did the PI group. The findings imply these two groups have different perpetuating mechanisms and treatment targets/needs.

Psychiatric comorbidity as a moderator of CBT response[1]. Dr. Edinger's group also conducted this study to determine if PI and CMI patients derive comparable benefits from CBT. Participants (70 men, 11 women; M_{Age} = 54.2 ±13.8 yrs.) met criteria for PI (n = 40) or CMI (n = 41). Most of the CMI patients met criteria for a depressive disorder (MDD or dysthymia; n = 16) or combat-related Posttraumatic Stress Disorder (n = 18). Patients were randomly assigned to either CBT or a sleep hygiene control therapy (SH) entailing lifestyle (e.g., limit caffeine) and environmental manipulations (e.g., keep bedroom dark) to enhance sleep. Each treatment consisted of 4 biweekly, 30- to 60-minute individual sessions with a study therapist.

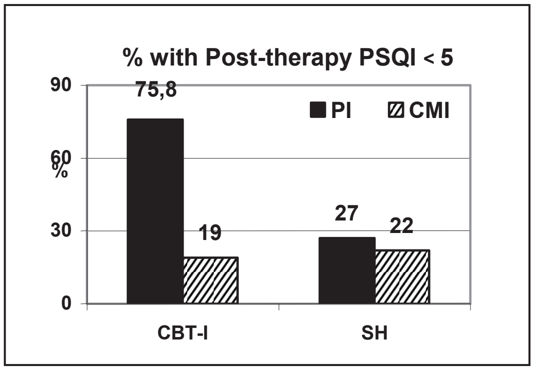

Comparisons of these groups using traditional quantitative sleep parameters (e.g., TST, SOL) suggested no consistent differences in their relative CBT responsiveness. However when more global measures of the overall insomnia syndrome were considered, the PI group was more responsive to CBT than the CMI group. For example, on the Insomnia Symptom Questionnaire, an instrument designed to assess changes in both daytime and nighttime symptoms, CBT-treated PI patients displayed a much larger treatment effect size (.87) than did CBT-treated CMI patients (.34). Moreover, 75.8% of the CBT-treated PI patients achieved normal scores on the Pittsburgh Sleep Quality Index by the end of treatment, compared to only 19% of the CBT-treated CMI patients (see figure). These two instruments most closely approximate the global insomnia disorder assessment provided by the Insomnia Severity Index, which will serve as the primary outcome for the project proposed. Thus, we believe these data suggest BT-treated CMI patients will have a lower ISI measured remission rate following first-stage treatment than will BT-treated PI patients.

c.3 Subjects

Selection Criteria. We will recruit adults (aged 21 and older) with chronic insomnia from the community and from outpatient medical and mental health clinics. Inclusion criteria will be broad to obtain results widely generalizable to the insomnia patient population commonly seen in practice. The inclusion criteria are: (a) a complaint of persistent (i.e., ≥ 1 month) difficulties initiating or maintaining sleep despite adequate opportunity for sleep; (b) a sleep onset latency or wake time after sleep onset > 30 minutes 3 or more nights per week during two weeks sleep diary monitoring; (c) an Insomnia Severity Index (ISI) score > 10 indicating at least "mild" insomnia; and (d) a score ≥ 2 on either the interference or distress item of the screening ISI, indicating the insomnia causes significant distress or impairment in social, occupational, or other areas of functioning. These criteria represent those provided in the DSM-IV-TR[87], Research Diagnostic Criteria[3] and the International Classification of Sleep Disorders[4], and will ensure a sample with clinically relevant insomnia.

Exclusion criteria will be minimal to retain a broadly representative sample that includes patients with primary and insomnia comorbid to a psychiatric disorder. Likewise, individuals with a comorbid medical condition will be excluded only if the medical condition is life-threatening or would contra-indicate using study medications. Exclusion criteria are (a) an untreated psychiatric disorder (e.g., major depression) as these conditions have specific treatments and it would be inappropriate not to offer those treatments; (b) a lifetime diagnosis of any psychotic or bipolar disorder as sleep restriction and medications for insomnia may precipitate mania and hallucinations; (c) an imminent risk for suicide; (d) alcohol or drug abuse within the past year, since BzRAs are cross-tolerant with alcohol; (e) terminal or progressive physical illness (e.g., cancer, COPD), or neurological degenerative disease (e.g., dementia); (f) current use of medications known to cause insomnia (e.g., steroids); (g) sleep apnea (apnea/hypopnea index > 15), restless legs syndrome, periodic limb movement during sleep (PLMS with arousal > 15 per hour), or a circadian rhythm sleep disorder (e.g., advanced sleep phase syndrome); (h) habitual bedtimes later than 2:00 AM or rising times later than 10:00 AM; (i) consuming > 2 alcoholic beverages per day on a regular basis.

Individuals using sleep-aids (prescribed or over-the-counter) will be included if they are willing and able to discontinue medications at least 2 weeks before baseline assessment. Participants using alcohol as a sleep aid or alcohol after 7:00pm on a regular basis will be required to discontinue this practice at least two weeks prior to baseline assessment. Individuals using psychotropic medications (e.g., anxiolytics, antidepressants) will not be automatically excluded from the study. Those on stable dosages (for at least three months) of SSRI or SNRI medications and who show at least partial remission (via SCID) from their mood or anxiety disorder will be accepted in the study if they meet the selection criteria above. Patients using TCAs, MAOIs, or atypical antidepressants will be excluded even if in remission as the effects of these medications on sleep might confound interpretation of the findings. We will impose similar standards for those with MDD, dysthymia, panic disorder, phobia, and GAD. We realize that some decisions about enrollment may not always be easy to make, but we will rely on all available data and a consensus approach to guide our clinical decision making process.

Recruitment/Participant Flow. Participants will be recruited at two sites (Duke and Laval Universities), each of which has an active insomnia research program with proven infrastructures for clinical trials. We will recruit participants through media advertisements, flyers distributed in outpatient clinics, and letters to primary care physicians and mental-health clinicians. We have ongoing referrals from these sources at each study site, including many with difficult forms of insomnia comorbid with psychiatric conditions. We recognize that CMI patients are relatively more prevalent than PI sufferers, and our study aims are designed to determine the optimal treatment(s) for such individuals. We will use a targeted recruitment approach so that roughly 60% (n = 192) of the 320 patients enrolled will have CMI. Given our usual mix of clinic and research referrals, we are confident the CMI patients enrolled will comprise a mixture of anxiety disorders (GAD, panic, phobia, PTSD) as well as mood disorders (e.g. dysthymia) other than major depression so our study results should add to and compliment ongoing NIMH funded trials focused exclusively on CMI in MDD. Based on our previous trials, we expect 400-500 individuals to inquire about the study at each site during the 48 months of enrollment. Approximately 40%-50% should meet study criteria so 3-4 participants are enrolled monthly at each site. We expect that attrition will be limited since study participants will be provided free ongoing treatment and financial incentives. Recent experiences with trials involving behavioral and pharmacological insomnia therapies at both sites have shown attrition rates in the 10% to 20% range. Should attrition be higher than expected, we are prepared to enroll 10-20 additional subjects per site in this trial to maintain adequate statistical power.

c.4 Sleep-Wake Monitoring

Polysomnography. Study participants will undergo standard nocturnal polysomnographic (PSG) monitoring for screening and outcome assessment. The initial PSG will serve to detect sleep disorders that would lead to exclusion (see selection criteria, c.3) and to provide an initial baseline night for those enrolled. Patients who meet criteria will complete additional PSG recordings at various times to assess outcomes (see Table C.5). PSG monitoring will be conducted in the sleep laboratories according to standard procedures with regard to montage and sampling rate (256 Hz), and bedtimes and rising times. Patients randomized to medication conditions will take their study medication (zolpidem or trazodone) at the time of sleep studies. Participants will undergo PSG on their usual dosages of allowed psychotropics (e.g., SSRI) and other medications (e.g., antihypertensive agents) since these medicated individuals will have active insomnia despite using medications. PSG recordings will be scored, blind to treatment assignment, in 30-second epochs using standard scoring criteria[88] for sleep staging and characterization of sleep-associated events (e.g., apneas). The initial screening/baseline PSG will be scored at the site where it is recorded to ensure a timely decision regarding study inclusion. All remaining PSG records will be scored centrally at the Duke site by trained

technologists under the supervision of Dr. Krystal, an experienced, board-certified polysomnographer.

IVRS Sleep Diary System. Subjective estimates of sleep and wake times will be obtained daily using an interactive telephone voice response system (IVRS). Participants will phone the IVRS system each morning and report the following information about their previous night's sleep: bedtime, sleep onset latency, number and length of nocturnal awakenings, time of final waking, rising time, ratings of sleep *quality* and *restedness* upon arising. Additional questions will query caffeine, alcohol, and sleep medication use. The IVRS program will automatically record a time and date stamp to verify when the data were entered. IVRS sleep diaries will be obtained for 2-weeks at baseline and at each subsequent assessment period.

c.5 Measures

Screening Instruments. Two structured interviews will be used. The *Duke Structured Interview for Sleep Disorders* (DSISD; Appendix II)[89], is an instrument developed by Dr. Edinger and colleagues to assist in ascertaining DSM-IV-TR[87] and International Classification of Sleep Disorders (ICSD-2)[4, 90, 91] sleep disorder diagnoses. This instrument has acceptable reliability and discriminant validity[1]. In addition, the *Structured Clinical Interview for DSM-IV* Axis I Disorders (SCID)[92] will be used to classify enrollees as having primary or comorbid insomnia and to identify study candidates with disorders (e.g., Bipolar Disorder) leading to exclusion. In addition to widespread use in clinical research, the SCID is supported by extensive reliability and validity data. All interviews will be audio taped and reliability checks will be conducted on 15% of them. We will administer the *Folstein Mini-Mental Status Exam* (MMSE)[93] to exclude those with cognitive deficits (MMSE score $\leq$ 27) that make them unable to give informed consent or fully participate in an interactive treatment.

Outcome Measures. The primary outcome metric will be the proportion of individuals achieving remission by producing scores $\leq$ 7 the Insomnia Severity Index (ISI) [32]. The ISI is a 7-item self-report questionnaire that provides a global measure of perceived insomnia severity based on several indicators (e.g., difficulty falling or staying asleep, satisfaction with sleep, degree of impairment with daytime functioning). The total score ranges from 0-28: 0-7 (no clinical insomnia), 8-14 (sub threshold insomnia), 15-21 (insomnia of moderate severity), and 22-28 (severe insomnia). The ISI has been validated[84] and has proven sensitive to therapeutic changes in several treatment studies of insomnia[69, 94].

We will track a number of secondary outcomes. Among these are measures of sleep/insomnia status including: sleep onset latency (SOL), wake time after sleep onset (WASO), total sleep time (TST), sleep efficiency (SE) taken from both sleep diaries and PSG. Diary ratings of sleep quality and feeling rested upon arising will also be obtained. Patients will complete the *Pittsburgh Sleep Quality Index (PSQI)*[95] at various time points to reflect their changes in overall sleep quality. The PSQI is widely used in clinical research and has validated cut-off scores to optimize sensitivity and specificity as a measure of insomnia[96, 97] A *clinical global improvement (CGI)*[98] rating will be completed by a blinded rater to provide a measure of insomnia severity and intervention effects. Despite some limitations about reliability and validity[99], the CGI will serve as a useful comparator with other insomnia trials. Finally the blinded rater will interview participants at the conclusion of their study involvement using the DSISD to determine whether they continue to meet RDC for insomnia.

To reflect treatment-related changes in daytime function, participants will complete the *Multidimentional Fatigue Inventory (MFI)*[100], the *SF-36 Health Survey (SF-36)*[101] and the insomnia adaptation of the *Sheehan Disability Scale (SDS-I)*[102]. The well-validated[100] 20-item MFI assesses several dimensions of fatigue (e.g., physical, mental) and has been used in studies of chronic pain[103], cancer [104] and depression [105]. The SF-36 is a quality of life measure that comprises eight scales (e.g., Physical functioning, Bodily Pain, Vitality) and 2 summary measures (Physical Health and Mental Health)[106, 107]. The SDS-I measures impairment in three major areas of functioning: work, social life/leisure activities, and home life/family responsibilities. It has been widely used in clinical trials for anxiety and depressive disorders and, more recently, in insomnia trials[108].

Changes in mood status will be assessed by including such well-validated and widely used measures as the *Beck Depression Inventory-II (BDI-II)*[109], *State-Trait Anxiety Inventory (STAI)*[110] and the *Beck Anxiety Inventory (BAI)*[111]. These instruments have well-established psychometric properties and have been used extensively in clinical research including studies of insomnia [112].

Adverse events (AE) monitoring will be achieved by using the *Systematic Assessment for Treatment Emergent Events (SAFTEE)* a reliable and valid instrument for assessing AEs related to study treatments.[113, 114] Finally, we will use an amended version of the *Therapy Evaluation Questionnaire* (appendix III) to assess treatment credibility, acceptability and patient satisfaction[115].

c.6 Procedures

Study candidates will undergo a multi-level screening. After an initial telephone screening, they will

complete: (a) the DSISD[89], SCID[92], and MMSE; (b) a medical history, physical exam, and laboratory tests (e.g., CBC, electrolytes, thyroid function tests, ECG); (c) a qualifying ISI; (d) 2 weeks of the IVRS sleep diary. Clinical and laboratory data will be reviewed on an ongoing basis in order to ascertain patients' suitability for the study.

Table C6. Time Course of Assessment and Study Procedures

MEASURES/TIME	Screen	Baseline	Treatment 1		Treatment 2		Follow-up (months)			
	- 4 weeks	-2 weeks	WKs 1-5	Wk # 6	Wks 7-11	Wk #12	3	6	9	12
**Telephone Screening**	X									
**Clinical evaluation of Insomnia – DSISD+RDC**	X									
**SCID/Psychological Screening**	X			X		X		X		X
**Medical History/Physical Exam**	X			X		X		X		X
**Drug/Alcohol Screen**	X			X		X				X
**Polysomnography - # of nights**		2		2		2				
**Sleep Diary**	2	2	X	X	X	X	X	X	X	X
**Insomnia Severity Index & Pittsburgh Sleep Quality Index**	X	X	ISI	X	ISI	X	X	X	X	X
**CGI + RDC assessment**			CGI	X	CGI	X	X	X	X	X
**Psychological Measures - BDI, STAI, BAI**		X		X		X	X	X	X	X
**Daytime Functioning – SF-36, MFI, SDS-I**		X		X		X	X	X	X	X
**Adverse Events Assessment – SAFTEE**		X		X		X	X	X	X	X
**Treatment credibility, preference, satisfaction**		X	Wk 1	X	Wk 7	X		X		X

Study candidates who meet selection criteria will complete PSG to rule other sleep disorders (e.g., apnea and PLMS). In the absence of such disorders during the first PSG night, a second consecutive PSG will be conducted for baseline purposes. Participants will then complete baseline assessment: (a) 2 weeks of nightly IVRS sleep diary monitoring; (b) self-rated questionnaires (c) clinician-administered assessments (ISI, CGI, RDC & SAFTEE adverse events). Participants will then be randomized to BT (n = 160) or MED (n = 160) treatments stratifying by gender, age, and psychiatric comorbidity. The latter classification will consider RDC and SCID criteria to determine whether patients have primary insomnia (PI) or comorbid insomnia (CMI).

During initial treatment with BT or MED, participants' ISI scores obtained at weeks 5 and 6 of treatment will be examined. Those who reach insomnia remission (ISI < 8) will receive no more provider contact but will continue their first-stage therapy independently for the next 6 weeks. Those who do not remit (ISI ≥ 8) will be encouraged to accept second stage therapy. Those who accept a second treatment will be randomized to an alternate insomnia therapy over the ensuing 6 weeks. Non-remitters who decline second-stage therapy will end their study participation at this point. During this second treatment phase, all participants will continue to collect nightly sleep diary data and complete the ISI (weekly) and other outcome questionnaires (at week 12), whether they receive a second therapy or not. Those receiving second-stage therapy will complete two PSG nights at week 12. Subsequently, all participants will enter follow-up wherein they will be contacted on a monthly basis for adverse event monitoring. Most such contacts will be by phone, but in-person visits will occur at months 3, 6, 9 and 12, when participants will be asked to complete additional IVRS sleep diaries (2 weeks), outcome questionnaires, and the SAFTEE. At the 12-month follow-up, a blinded clinician will use the DSISD to determine if participants meet RDC criteria for insomnia disorder (see Table C6 for sequence of procedures).

c.7 Treatments

Psychological Therapy. The first-stage psychological therapy will be Behavioral Therapy (BT), comprised of sleep restriction[116] and stimulus control therapies[117]. These well-established strategies are designed to strengthen homeostatic sleep drive, consolidate sleep via reducing time in bed, establish a regular sleep schedule, and curtail sleep-incompatible behaviors. Sleep hygiene education also will be included in BT to address lifestyle (e.g., caffeine use) and environmental factors (e.g., light, noise) that affect sleep.

The second-stage psychological treatment will consist of Cognitive Therapy (CT). CT is aimed at altering sleep-disruptive and mood-disturbing cognitions that exacerbate the vicious cycle of insomnia. Such cognitions are typically related to thoughts and beliefs about unmet sleep requirements (e.g. "I must have 8 hours of sleep every night") and potential insomnia consequences (e.g., "insomnia is necessarily detrimental to health and productivity"). Perpetuating mechanisms such as excessive self-monitoring and worries are also prime targets for CT. CT will follow standard procedures to identify and alter these sleep-interfering cognitions via recording automatic thoughts, Socratic dialogue, constructive worry, and behavioral experiments[118, 119].

Our decision to use BT as first-stage and CT as second-stage therapy (rather than a full CBT package introduced at once) was guided by several factors. First, BT is a brief intervention that can be implemented in most clinical settings, thus, enhancing its transferability to clinical practice. In contrast, CT is more time consuming and may not be essential for many with insomnia. Yet, because of its unique features in targeting some cognitive perpetuating mechanisms (e.g., worries, ruminations, low mood, self-monitoring) shared by insomnia and some comorbid psychiatric disorders (e.g., anxiety, depression), use of CT separately from the BT component will provide an opportunity to evaluate its unique contribution to outcomes among those with and without comorbid psychiatric disorders. We expect CMI patients to have a better response to this cognitive intervention. This innovative feature is also likely to make the switch within the psychological treatment modality (i.e., from BT to CT) more equivalent conceptually to the switch within the pharmacotherapy modality (i.e., from zolpidem to trazodone). These second stages therapies may have a broader mechanism of action in addressing sleep and mood symptoms relative to BT and zolpidem that target only sleep.

Medication (MED). The first-stage pharmacological treatment involves zolpidem, 5 to 10 mg, taken nightly at bedtime for the 6-week duration of first stage treatment. The primary factor leading to the choice of zolpidem as a first-stage therapy is that it has consistently been among the most commonly prescribed medications for treating insomnia and its efficacy as an insomnia therapy is well-documented[43, 44, 120]. It also has efficacy for treating the insomnia of depressed patients treated also with antidepressant medications[121]. The primary concern about zolpidem is that it has established efficacy mainly for sleep onset difficulties but only limited evidence showing its benefit for sleep maintenance problems[121]. Yet, we believe that the other considerations outweigh this one. Despite reports highlighting its behavioral side effects, including sleep eating, sleep walking, and other complex behaviors, these events are not specific to zolpidem, and are at least partly related to additional factors (e.g. excessive doses, combination with alcohol or other medications, and sleep deprivation). Enrollees will be advised about each of these. Zolpidem is preferable to alternatives such as eszopiclone and temazepam, which have longer half-lives and greater potential for morning sedation and shorter-acting drugs such as zaleplon and ramelteon, which are less likely to reduce WASO.

Participants will start with an initial dose of 5 mg, in consideration of our participants' age range and the desire to minimize potential side effects. The physician may adjust the dosage between 5 mg and 10 mg depending on the patient's age, therapeutic response, and side effects. Support and encouragement to comply with the prescribed medication regimen will be provided, but no BT or CT interventions will be allowed during these sessions. The physician will use a treatment manual specifically developed for this study, designed to standardize administration of the medication. All medications will be dispensed by the pharmacy at each site.

The second-stage pharmacotherapy will consist of trazodone (50-150mg), taken 30 minutes before bedtime. We chose trazodone because it is also among the most commonly prescribed medications for insomnia in clinical practice[43, 44]. It has shown efficacy for insomnia occurring with major depression, and it is the most commonly administered treatment for this problem by psychiatrists[45-47]. Admittedly very few studies have been conducted with this medication and it has only short-term (e.g. one week) efficacy in primary insomnia[122]. Nonetheless, testing trazodone's efficacy for insomnia is an important endeavor. As noted in the 2005 NIH state-of-the science conference on the manifestations and management of chronic insomnia summary report: "…, the antidepressant trazodone is now the most commonly prescribed medication for the treatment of insomnia in the United States. In short-term use, trazodone is sedating and improves several sleep parameters. All antidepressants have potentially significant adverse effects, raising concerns about the risk–benefit ratio. Moreover, there is a need to establish and communicate to prescribers dose-response relationships for all of these agents"[85].

c.8. Treatment Implementation/monitoring

First-stage and second-stage treatments. The first-stage psychological treatment, BT, will be administered in the context of four, individual, 45-min sessions led by a trained therapist (clinical psychologist) spread over the 6-week period (i.e. Weeks 1, 2, 4 and 6). The first stage medication therapy, zolpidem, will be administered by a physician in the context of four, similarly scheduled individual, 20-min consultation sessions over the same 6-week period. Participants who do not remit with first-stage therapy will be encouraged to continue with second-stage therapy also implemented in the context of four, individual, consultations visits. Participants in full remission after first-stage therapy will continue on their initial treatment regimen and receive no further therapy visits, except for monthly adverse event monitoring via phone and the regularly scheduled follow up visits. For example, those treated with medication will continue receiving the study medication and BT participants will be instructed to continue using the procedures they learned during the initial treatment phase. Our past studies suggest roughly 40% of enrollees will remit with initial treatment and 60% will continue

suffering clinically significant insomnia making them eligible to continue into a second-stage therapy.

Therapists and treatment manuals. Trained clinicians will deliver treatments guided by therapy manuals. Clinical psychologists will administer BT and CT, and physicians will administer medications. All therapists will receive training at a central site (Laval University) and will meet minimal competency criteria prior to treating study participants. The BT/CT manual will be modeled after published therapy manuals[32, 119, 123]. The pharmacotherapy manual will be based on those used in our previous studies of insomnia medication.

Treatment fidelity checks. All therapy sessions will be audio-taped and a random portion (15%) will be rated with standard checklist by blinded raters for the presence of essential ingredients of a given treatment and for the absence of proscribed treatment instructions. Clinicians who fail to meet criterion will receive additional training and, if unable to meet minimal competency criteria, they will be replaced. Participant's performance will be evaluated using periodic drug/alcohol screens, therapist ratings of BT and CT adherence (e.g., time spent in bed) and/or use of study medications (e.g., frequency and dosage), and weekly pill count.

Drug tapering. At the end of treatment, participants on medication will receive a last medication supply and a written withdrawal schedule (designed by study physician) for the subsequent 2-3 weeks. They will be informed of possible rebound insomnia and instructed not to discontinue medication abruptly. The time individuals require for discontinuing medication may vary, yet a 2-3 week taper should be adequate for most. A similar schedule will be followed for those using trazodone during second stage treatment. Those staying on medication through follow-ups will use a similar discontinuation schedule at the end of the 12-month follow up. Those who wish to continue medication will be referred to their primary care physician for further follow-ups.

c.9 Data Management and Analyses

This section was revised based on the useful critiques from the Summary Statement. For instance, expected attrition rates were revised upward and better justification is provided based on previous studies using similar treatment conditions (and no placebo control). Strategies to examine the impact of missing data, specifically MAR or NMAR, are specified and power estimates were recomputed accordingly. Linear mixed models were replaced with weighted generalized estimating equations when appropriate (Aim 2 analyses) and sensitivity analyses are planned to capture the uncertainty of some parameters (attrition, covariance structure). Statistical analyses will be performed and reported according to the CONSORT guidelines[124]

Randomization. Equal numbers of patients will be randomly assigned to the two stage-one therapies (n = 160 to BT, n = 160 to zolpidem), stratified by clinical site. Random block sizes of two and four will be used to insure balance while minimizing the probability of identifying the assigned condition. Assignment to treatment will be determined by a computer-generated random allocation schedule. Sealed, consecutively numbered envelopes with treatment assignment will be used to conceal randomization. Once a patient is eligible for study inclusion, an envelope with a pre-determined group assignment will be opened by the study coordinator. After completing stage one therapy, only patients who do not remit and consent to enroll in stage two will be randomized to a second stage treatment: (1) those not remitting with BT will be equally assigned to either zolpidem or CT, and (2) those who do not remit with zolpidem will be assigned to either BT or trazodone.

Data Management. We will use direct data entry tools (tablet PCs and IVRS diaries) when feasible to minimize data collection errors and facilitate data management. Paper-based data collection will be entered at each site in an Access database by two independent assistants. Dr Ivers (statistician) will revise data periodically to identify missing or incoherent data. Weekly reports about completion rates will be mailed to each PI for study monitoring. Computerized data entry and ongoing revision of all data collected on-site will reduce missing data. We will investigate missing data patterns to ascertain if data are missing completely at random (MCAR), at random (MAR) or not at random (MNAR)[125]. Guided by experts on longitudinal data analysis with missing data[126] and a recent review of 48 RCT datasets from 25 NDA submissions of neuropsychiatric drugs[127], our primary analyses will use statistical models that are robust to MCAR and MAR patterns. However, we will perform sensitivity analyses for MNAR considering specific reasons for attrition if needed[128]. No data imputation will be performed and all available observations will be included in inferential analyses.

Data Exploration. Descriptive statistics will be used to summarize all study variables. We also will construct plots of longitudinal outcome variables to understand their general trends over the study period. We will examine all variables to determine if parametric distributional assumptions are valid. Because the inferences in our analyses are robust to departure from normality, no transformations will be made if the variables are approximately normal. Sociodemographic, psychiatric, medical, and sleep characteristics will first be described using central tendency and dispersion indices for continuous variables, and frequency distributions for nominal data. To confirm the balanced randomization, treatment conditions will then be

compared using appropriate parametric or non-parametric tests.

Primary Outcome Analyses. All primary outcome analyses will be conducted based on the Intention-to-Treat (ITT) principle. Accordingly, all patients with at least one post of baseline data will be included in these analyses. To control for possible site effects, clinical site will be included as a main effect and the inclusion of a clinical site interaction with other main effects (when appropriate) will be investigated for significance.

Hypothesis 1a. The proportion of patients achieving remission with first-stage therapy and sustain remission through follow-up will be higher among those receiving BT than among those receiving zolpidem. A standard logistic regression will be used to compare the probability of short-term (after Stage I treatment) and sustained remission (primary outcomes) between BT and zolpidem conditions. Sustained remission is defined as an ISI score < 7 observed after Stage 1 therapy and maintained at (12 weeks and the 12-month follow-up).

Hypothesis 1b. A lower proportion of the CMI patients will achieve remission with first stage therapies than will those with PI. A similar logistic regression will be used to compare the probability of short-term (after stage one treatments) and sustained remission (primary end point) according to the presence of psychiatric comorbidity. This effect will be investigated as a moderator of the impact of stage one treatment on remission. The significance of a moderator is presumed by a significant moderator X treatment interaction[129]. To investigate the moderating effect of psychiatric comorbidity, this variable and its interaction with treatment will be added as fixed effects to the logistic regression model. A significant interaction then will be explored using simple effects to test if CMI is associated to poorer acute and long-term outcomes for each stage one therapy.

Hypothesis 1c. Secondary outcomes (PSG, sleep diary, fatigue, mood, etc) will show greater improvements through treatment and follow-up for those receiving BT than for those receiving zolpidem. Comparisons between these therapies for secondary outcome measures will be performed using a mixed model approach[130], including a generalized mixed-effect regression models for binary variables (e.g., diagnosis for insomnia) and a normal mixed-effect regression models for continuous variables. In both modeling approaches, treatment, time and potential confounding factors will be included in the models as fixed main effects. The models will also include random effects for intercept and slope (patient by time) for each subject.

Hypothesis 2a. The insomnia remission rate after second-stage treatment for all conditions combined will be 20% higher than with first-stage treatment (i.e., increment from 40% to 60%). Since participation in the second randomization is conditional upon not remitting with first-stage therapies, generalized estimating equations models (GEE) will be used to test whether overall remission rate significantly increased. GEE is a semi-parametric statistical approach that provides robust empirical estimates by avoiding full specification of the joint distribution of outcomes. The weighted GEE approach[131] will be preferred in this trial to take into account the missing data pattern (MCAR or MAR).

Hypothesis 2b. Of all patients who enter second-stage treatment, a greater proportion who switch modalities (i.e., BT→zolpidem or zolpidem→BT) will achieve remission and sustain it through follow-up than will those staying within a treatment modality. To maximize degrees of freedom, weighted GEE models will be used to compare remission rates after 12 weeks of treatment and sustained remission after 12 months, according to two main effects: (a) having received BT or zolpidem during Stage I and, (b) having switched treatment modality or not during Stage II. These main effects and their interactions will capture the partial effect of each treatment combination while accounting for relevant patient trajectory (data from Stage I & Stage II therapies). Other main fixed effects (e.g., time) and potential baseline covariates will be included. Weights will be computed for each case after specifying a drop-out model estimated from the observed drop-out patterns.

Hypothesis 2c. CMI patients who enter second-stage treatment will show a higher remission rate with treatments that target sleep and mood symptoms (e.g. CT and trazodone) than with treatments targeting primarily sleep (BT and zolpidem). This added therapeutic effect will be higher in CMI than in PI patients. Two tests will be of primary interest within the full factorial (comorbidity x conditions x time) weighted GEE model: (a) an a priori contrast to examine remission rates after stage two treatment according to whether CMI patients received the "mood addressing" sequences (BT→CT or zolpidem→trazodone) or the "non-mood" sequences (BT→zolpidem or zolpidem→BT); (b) a comorbidity x treatment interaction will be used to compare whether the added therapeutic effect obtained by addressing mood symptoms is higher for CMI than for PI patients. Significant interactions will be examined with simple effects to test whether temporal changes observed for each subgroup during second-stage therapies are significant. Other fixed effects such as first-line therapy and potential baseline covariates will be included.

Hypothesis 2d. Secondary outcomes will show response patterns consistent with Hypotheses 2a-c. Analyses related to these hypotheses will be based on the scale of measurement. When the outcomes are

assessed on a binary scale, identical analyses will be performed. When the outcome are assessed on a continuous scale, weighted GEE will be specified with a normal distribution and an identified link function.

Sample Size and Detectable Effect Size. All sensitivity power analyses were computed following procedures outlined by Stroup[132] for mixed models and Dahmen et al.[133] for weighted GEE models, and were based on standard conditions: a two-sided alternative hypothesis, 80% power, and a type I error rate of 5%. Based on studies conducted by our group [2, 134, 135], and recent reviews of the literature on this topic[136], attrition rates of 10% for BT/CT conditions and 20% for zolpidem/trazodone conditions during stage one and two therapies, and 10% at 12-months follow-up were used as attrition estimates in the computation of detectable differences. We recognize this limitation, but lack of preliminary data on primary and secondary outcomes precluded computation and further inclusion of random outcomes variance in the power computations[137].

Hypothesis 1a: Assuming a one-tailed directional hypothesis (BT > zolpidem for remission), our sample size of 320 will give a standard power to detect a difference of 14.7% in sustained remission between BT and zolpidem.

Sensitivity analyses for Hypothesis 1a

Sample size per group	120	140	160	180	200
Detectable difference in remission rate	17.2%	15.9%	14.7%	13.9%	13.2%

Hypothesis 1b: Since this hypothesis is tested using a similar analytic strategy as H1a, very similar detectable differences are observed in power computations and thus are not reported here.

Sensitivity analyses for Hypothesis 1b

Sample size per group	120	140	160	180	200
Cohen's f (small = 0.10)	.076	.071	.066	.062	.059
Detectable difference in SOL (min.)	4.8	4.5	4.2	3.9	3.7
Detectable difference in WASO (min.)	5.5	5.2	4.8	4.5	4.3

Hypothesis 1c: A sample size of 320 (effective sample = 272) would give a standard level of power to detect a small effect size (Cohen *f* = 0.066) on the condition (BT vs. zolpidem) X time (baseline vs. Post-I) interaction. Translated into clinically relevant units (based on variability estimates from Morin et al., 2009), this sample size would allow the detection of a difference of 4.2 minutes of SOL and 4.9 minutes of WASO between temporal changes observed in the BT condition vs. those observed in the zolpidem condition.

Hypothesis 2a: A sample size of 320 (effective sample at Post 2 = 234 after taking into account attrition) will give a standard power to detect a increment of 8.2% in remission rate between Post I and Post II assessments, assuming a working correlation of 0.50.

Sensitivity analyses for Hypothesis 2a

Sample size per group	120	140	160	180	200
Detectable difference in remission rate	10.0%	9.0%	8.2%	7.2%	6.4%

Hypothesis 2b: A sample size of 320 (effective sample at Post 2 = 139 after attrition and after the second randomization on about 60% of the remaining sample) will give a standard level of power to detect a 28.6% difference in remission between patients who switched treatments and those who did not, and sufficient power to detect an increase in remission rate of 20.5% for patients who switched treatments.

Sensitivity analyses for Hypothesis 2b

Sample size per group	120	140	160	180	200
Detectable difference in remission rate for interaction effect	32.3%	30.7%	28.6%	25.3%	22.8%
Detectable difference in remission rate for time effect (switchers)	25.3%	22.7%	20.5%	18.1%	16.1%

Hypothesis 2c: Since this hypothesis is tested on the same sample size than H2b (effective sample at Post 2 = 139 after attrition and after the second randomization on about 60% of the remaining sample), similar levels of power and minimally detectable differences are expected. Results are thus not reported here.

Hypothesis 2d: This hypothesis is tested using the same analytical procedure as for H2a-H2c but it is completed with continuous data wherein baseline data can be included. These tests are much more powerful. For a sample size of 320, all detectable Cohen's f values are found to be less than 0.10 (e.g., a difference of 7.5 min on SOL between switchers or non-switchers, or between mood-addressing or not modalities).

**Note*. Reprinted from "Effectiveness of Sequential Psychological and Medication Therapies for Insomnia Disorder: A Randomized Clinical Trial", by Morin CM, Edinger JD, Beaulieu-Bonneau S, et al., 2020, JAMA Psychiatry, 77(11):1107-1115.
