# Supplementary material for: Effect of Psychological and Medication Therapies for Insomnia on Daytime Functions: A Randomized Clinical Trial
Source: JAMA Netw Open. 2023 Dec 28;6(12):e2349638. doi: 10.1001/jamanetworkopen.2023.49638 (PMC10755607; doi:10.1001/jamanetworkopen.2023.49638)
Supplement: Supplement 2. — eTable. Significance Level of Between-Sequence Statistical Comparisons [file jamanetwopen-e2349638-s002.pdf]

Supplementary Online Content

Morin CM, Chen SJ, Ivers H, et al. Effect of psychological and medication therapies for insomnia on daytime functions: a randomized clinical trial. *JAMA Netw Open.* 2023;6(12):e2349638. doi:10.1001/jamanetworkopen.2023.49638

eTable. Significance Level of Between-Sequence Statistical Comparisons

This supplementary material has been provided by the authors to give readers additional information about their work.

© 2023 Morin CM et al. *JAMA Network Open.*

eTable. Significance Level of Between-Sequence Statistical Comparisons

Outcome	Post1 (b)	Change (b-a)	Post2 (c)	Change (c-b)	FU-3m	FU-12m (d)	Change (d-c)
STAI-trait	P = .06 [a]	P = .02*	P = .53	P = .005*	P = .41	P = .57	P = .59
BDI-II	P = .85	P = .38	P = .08 [a]	P = .08 [a]	P = .24	P = .08 [a]	P = .74
MFI	P = .60	P = .79	P = .42	P = .72	P = .78	P = .96	P = .55
WSAS	P = .61	P = .96	P = .19	P = .54	P = .50	P = .47	P = .20
SF36 Physical Health scale	P = .74	P = .17	P = .60	P = .99	P = .18	P = .61	P = .54
SF36 Mental Health scale	P = .49	P = .44	P = .008*	P = .001*	P = .17	P = .10	P =. 73

Abbreviations: BT, behavioral therapy; BDI-II, Beck Depression Inventory-II; CT, cognitive therapy; MFI, Multidimensional Fatigue Inventory; SE, standard error; SF-36, SF-36 Health Survey; STAI-Trait, State-Trait Anxiety Inventory.

* $P < .05$; [a] $P < .10$.

© 2023 Morin CM et al. *JAMA Network Open.*
