# Supplementary material for: Effect of Psychological and Medication Therapies for Insomnia on Daytime Functions: A Randomized Clinical Trial
Source: JAMA Netw Open. 2023 Dec 28;6(12):e2349638. doi: 10.1001/jamanetworkopen.2023.49638 (PMC10755607; doi:10.1001/jamanetworkopen.2023.49638)
Supplement: Supplement 3. — Data Sharing Statement [file jamanetwopen-e2349638-s003.pdf]

Data Sharing Statement

Morin. Effect of Psychological and Medication Therapies for Insomnia on Daytime Functions. *JAMA Netw Open*. Published December 28, 2023. doi:10.1001/jamanetworkopen.2023.49638

Data
Data available: Yes
Data types: Deidentified participant data
How to access data: The data will be made available upon publication to researchers who provide a methodologically sound proposal for use in achieving the goals of the approved proposal. Proposals should be submitted to cmorin@psy.ulaval.ca.
When available: With publication

Supporting Documents
Document types: None

Additional Information
Who can access the data: Researchers whose proposed use of the data has been approved.
Types of analyses: For any purpose.
Mechanisms of data availability: After approval of a proposal.
